# Cellulose‐Derived Functional Polyacetal by Cationic Ring‐Opening Polymerization of Levoglucosenyl Methyl Ether

**DOI:** 10.1002/anie.201908458

**Published:** 2019-10-25

**Authors:** Tapas Debsharma, Yusuf Yagci, Helmut Schlaad

**Affiliations:** ^1^ Institute of Chemistry University of Potsdam Karl-Liebknecht-Straße 24–25 14476 Potsdam Germany; ^2^ Department of Chemistry Istanbul Technical University Maslak 34469 Istanbul Turkey

**Keywords:** biomass valorization, cationic ring-opening polymerization, levoglucosenyl methyl ether, polyacetals, semicrystalline materials

## Abstract

The unsaturated bicyclic acetal levoglucosenyl methyl ether was readily obtained from sustainable feedstock (cellulose) and polymerized by cationic ring‐opening polymerization to produce a semicrystalline thermoplastic unsaturated polyacetal with relatively high apparent molar mass (up to ca. 36 kg mol^−1^) and decent dispersity (ca. 1.4). The double bonds along the chain can undergo hydrogenation and thiol–ene reactions as well as crosslinking, thus making this polyacetal potentially interesting as a reactive functional material.

## Graphical abstract

**From crops to CROP**: Cationic ring‐opening polymerization (CROP) of biomass‐derived levoglucosenyl methyl ether yields a semicrystalline thermoplastic unsaturated polyacetal. The double bonds can undergo hydrogenation and thiol–ene reactions as well as crosslinking, thus making this polyacetal potentially interesting as a reactive functional material.

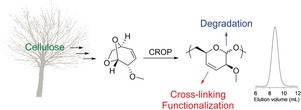

The exploitation of fossil‐based resources has given comfort and wealth to society at the expense of increasing atmospheric carbon dioxide concentration and other environmental hazards. The rise in carbon dioxide concentration increases carbohydrate concentration, thereby reducing the overall content of protein in plants.^1^ Moreover, the plastic industry is particularly dependent on fossil‐based resources that exist in limited amounts, and the produced non‐degradable plastics create many environmental problems. It is therefore important to move towards renewable feedstocks, valorization of biomass, and environmentally degradable systems.^2^ In this respect, biologically sourced polymers have been of interest among the scientific community to tackle the above‐mentioned problems.^3^

Cellulose, being the most abundant product of biomass on earth, is an attractive renewable, non‐edible resource for the production of many value‐added chemicals such as sugars, lactic acid, levulinic acid, or furans.^4^ Another molecule with relatively complex bicyclic structure that can be obtained through the pyrolysis of cellulose is levoglucosenone (**1**, Scheme 1).^5^ Nowadays, levoglucosenone is produced in industrial quantities (50 tons per year) by the Circa Group Ltd., Australia, and the derivative dihydrolevoglucosenone (Cyrene) has been launched as an environmentally friendly solvent to replace dipolar aprotic solvents like *N*‐methyl‐2‐pyrrolidone (NMP).^6^ Levoglucosenone is used for the synthesis of chiral therapeutic agents and molecules with fixed and known stereochemistry,^7^ however it has not yet entered the field of polymers.

**Scheme 1:**
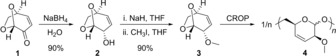
Synthesis of levoglucosenyl methyl ether **3**, starting from levoglucosenone **1** via levoglucosenol **2**, and polymerization through CROP to yield the polyacetal **4**.

Free radical or anionic polymerizations of **1** has only produced oligomers at best.^8^ Its alcohol derivative levoglucosenol (**2**, Scheme 1), on the other hand, was found to polymerize through ring‐opening olefin metathesis polymerization (ROMP) to yield an amorphous thermoplastic polyacetal.^8^ Levoglucosenol should, on a first glance, also polymerize via the acetal functionality through cationic ring‐opening polymerization (CROP). In fact, molecules with similar or related structures, that is, anhydrosugars^9^ and bicyclic ketals,^10^ have been polymerized successfully through Lewis acid‐catalyzed CROP. However, attempts to polymerize levoglucosenol (**2**) through CROP failed. We therefore decided to mask the hydroxy function of levoglucosenol by methylation to yield the levoglucosenyl methyl ether **3** (IUPAC name: 4‐methoxy‐6,8‐dioxabicyclo[3.2.1]oct‐2‐ene; Scheme 1). CROP of **3** would then give the linear unsaturated polyacetal **4** with the proposed chemical structure shown in Scheme 1, which is potentially degradable^11^ and could be further modified or crosslinked.^12^ It is worth being mentioned that **3**, like its precursor **2**,^8^ can also be polymerized through ROMP (preliminary data, not shown).

The overall synthetic procedure for the levoglucosenyl methyl ether **3** is shown in Scheme 1. Levoglucosenone (**1**) is reduced by sodium borohydride in water, and the resulting levoglucosenol (**2**) is then deprotonated with sodium hydride and methylated with methyl iodide to yield **3** (see the experimental procedures in the Supporting Information). Purification of **3** was achieved by distillation, and the overall yield was 81 %. The chemical structure of **3** was confirmed by nuclear magnetic resonance (NMR) spectroscopy and electrospray ionization mass spectrometry (ESI‐MS; see Supporting Information) to be (1*S*,4*S*,5*R*)‐4‐methoxy‐6,8‐dioxabicyclo[3.2.1]oct‐2‐ene (major isomer, 96 %). Notably, the synthesis of **3** is far less complicated and tedious than that of other sugar‐based monomers for ROP.^13^

First attempts to polymerize **3** involved the use of triflic acid (CF_3_SO_3_H, TfOH) and boron trifluoride etherate (BF_3_⋅OEt_2_). Polymerizations were conducted in dichloromethane (DCM) solution at room temperature or 0 °C for 24 h and were quenched with triethylamine; results are summarized in Table 1. TfOH appeared to be a very efficient initiator for the CROP of **3**. Monomer conversion (*x*_p_) reached more than 90 % under the chosen conditions, though a slightly higher molar mass polymer **4** [*M*_n_^app^=18.6 kg mol^−1^, *Ð*=1.4; by size exclusion chromatography (SEC)] was obtained at lower temperature. The attempted polymerizations of **1** and **2** with TfOH in DCM solution at room temperature failed; either no reaction occurred or yet unidentified organic compounds were produced.

**Table 1:** CROP of **3** in DCM solution with triflic acid or boron trifluoride etherate as initiator/catalyst (acid) for 24 h.

Entry	Acid	[**3**]_o_/[acid]	[**3**]_o_ [m]	*T* ^[a]^ [°C]	*x*_p_^[b]^ [%]	*M*_n_^app[c]^ [kg mol^−1^]	*Ð* ^[d]^
1	TfOH	200:1	4	25	92	15.1	1.4
2	TfOH	200:1	4	0	92	18.6	1.4
3	BF_3_⋅OEt_2_	200:1	2	24	2	–^[e]^	–^[e]^
4	BF_3_⋅OEt_2_	5:1	2	0	84	11.8	1.5

[a] Reaction temperature. [b] Monomer conversion, determined by ^1^H NMR spectroscopy. [c] Apparent number‐average molar mass, determined by SEC with polystyrene calibration. [d] Dispersity index, determined by SEC. [e] Not determined.

Polymer **4** was found to be soluble in DCM, chloroform, tetrahydrofuran (THF), and acetonitrile but insoluble in diethyl ether, dimethyl sulfoxide (DMSO), methanol, and water. Its chemical structure and optical activity were confirmed by NMR and circular dichroism (CD) spectroscopy (see Figure 1 and the Supporting Information). Importantly, the polymer chains contain exclusively one sequence isomer (as evidenced by the sharp singlet signals in the ^13^C NMR spectrum, Figure 1 b) and the double bonds were fully retained.

**Figure 1:**
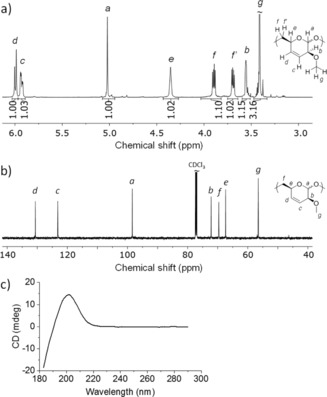
a) ^1^H NMR (600 MHz) and b) ^13^C NMR (150 MHz) spectra of polymer **4** (entry 2 in Table 1) in CDCl_3_, c) CD spectrum of 0.033 wt % solution of **4** in acetonitrile (*λ*_max_=202 nm).

Polymer **4** is a semicrystalline thermoplastic, showing a glass transition at around 35 °C and melting transitions at 40–120 °C, and is thermally stable up to around 220 °C, as determined by differential scanning calorimetry (DSC) and thermogravimetric analysis (TGA; see Figure 2 a and the Supporting Information). The semicrystalline nature of the polymer was also seen by polarized optical microscopy (POM; Figure 2 b). Furthermore, the polymer was found to degrade quickly, within several hours, in DCM solution in the presence of BF_3_⋅OEt_2_ and methanol as a nucleophile, as expected (see the Supporting Information).

**Figure 2:**
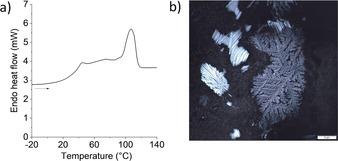
a) DSC 1^st^ heating curve (heating rate: 10 K min^−1^) of polymer **4** (entry 12 in Table 2) and b) POM image (crossed polarizers, scale bar=5 μm) of a polymer film after heating to 120 °C and slowly cooling down to room temperature (crystals started to form at ca. 57 °C).

BF_3_⋅OEt_2_ also catalyzes the polymerization, but rather high catalyst concentrations (20 mol % with respect to monomer) are required to attain high monomer conversion (entry 4 in Table 1). The need for a high loading of BF_3_⋅OEt_2_ can be explained by the fact that trace amounts of water are crucial to initiate the polymerization. This can be supported by previous studies demonstrating that BF_3_ etherate failed to polymerize trioxane in rigorous dry conditions.^14^ In addition, the achieved molar mass of the polymer is not linearly related to the amount of catalyst and no polymerization occurred at low catalyst concentration (entry 3 in Table 1). This is likely due to the equilibrium nature of the reaction between water and BF_3_⋅OEt_2_. A higher amount of BF_3_⋅OEt_2_ is needed to shift the equilibrium to the right‐hand side to produce protons as the initiating species (Scheme 2).

**Scheme 2:**
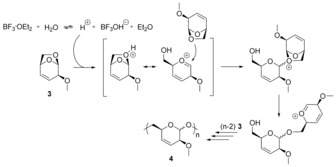
Equilibrium reaction of BF_3_⋅OEt_2_ with water to release protons and proposed pathway of proton‐initiated cationic polymerization of **3**.

The polymerization of **3** is believed to proceed via an oxonium ion through an active‐chain‐end mechanism (Scheme 2). The activation of the cyclic ether ring by acid catalyst leads to the opening of the bicyclic ring followed by stabilization of the anomeric carbocation via the formation of an oxonium ion. Successive attack of the monomer by OCH_2_, which is more nucleophilic than the competing OCH, should essentially lead to the formation of the polymer **4** with a thermodynamically favorable six‐membered ring structure.

Although TfOH was found to be the more effective initiator, BF_3_⋅OEt_2_ was easier to handle and therefore chosen as the catalyst for further screening experiments. Polymerizations of **3** with BF_3_⋅OEt_2_ were conducted at different monomer‐to‐catalyst ratios (10:1 to 10:3), monomer concentrations (3 or 4 m), reaction temperatures (−50 to 25 °C), and times (1.5 to 48 h); results are summarized in Table 2. The highest monomer conversion (97 %) and polymer molar mass (28.8 kg mol^−1^) were obtained with a high catalyst loading (30 mol %) at a temperature of −10 °C in DCM solution (entry 9 in Table 2). The polymer **4** exhibited a monomodal molar mass distribution with a dispersity of 1.4.

**Table 2:** Polymerization of **3** with BF_3_⋅OEt_2_ at different monomer‐to‐catalyst ratios, monomer concentrations, temperatures, and reaction times.

Entry	[**3**]_o_/ [BF_3_⋅OEt_2_]	[**3**]_o_ [m]	*T* ^[a]^ [°C]	*t* ^[b]^ [h]	*x*_p_^[c]^ [%]	*M*_n_^app[d]^ [kg mol^−1^]	*Ð* ^[e]^
1	10:1	3	−50	48	3	–^[f]^	–^[f]^
2	10:1	4	−50	48	9	18.6	1.3
3	10:1	3	−20	48	70^[g]^	23.9	1.3
4	10:1	4	−20	24	74^[g]^	25.3	1.3
5	10:1	3	−10	24	77	19.3	1.3
6	10:1	4	−10	24	87	21.2	1.4
7	10:2	3	−10	24	92	20.3	1.4
8	10:2	4	−10	24	94	21.7	1.5
9	10:3	4	−10	24	97	28.8	1.4
10	10:1	3	0	24	90	17.2	1.4
11	10:1	4	0	24	93	19.8	1.4
12^[h]^	10:1	4	0	24	97	22.2	1.4
13	10:1	4	25	1.5	92	21.8	1.5

[a] Reaction temperature. [b] Reaction time. [c] Monomer conversion, determined by ^1^H NMR spectroscopy. [d] Apparent number‐average molar mass, determined by SEC with polystyrene calibration. [e] Dispersity index, determined by SEC. [f] Not determined. [g] Reaction mixture turned solid (frozen) at the given monomer conversion, re‐liquefied upon the addition of solvent. [h] Final sample of the kinetic experiment.

A study of the kinetics of the polymerization of **3** (Figure 3 a) revealed that the monomer was quickly consumed within less than 1 hour, following pseudo‐first‐order kinetics, but leveled off thereafter. The apparent number‐average molar masses (*M*_n_^app^) increased constantly to around 36 kg mol^−1^ (*x*_p_=78 %) but decreased at very high monomer conversions (*x*_p_>90 %, Figure 3 b), probably due to chain‐transfer and back‐biting reactions (which are often observed for cationic polymerizations of cyclic ethers^15^) producing new growing chains.^16^ Nevertheless, all polymer samples showed a monomodal and fairly narrow molar mass distribution (Figure 3 c and the Supporting Information).

**Figure 3:**
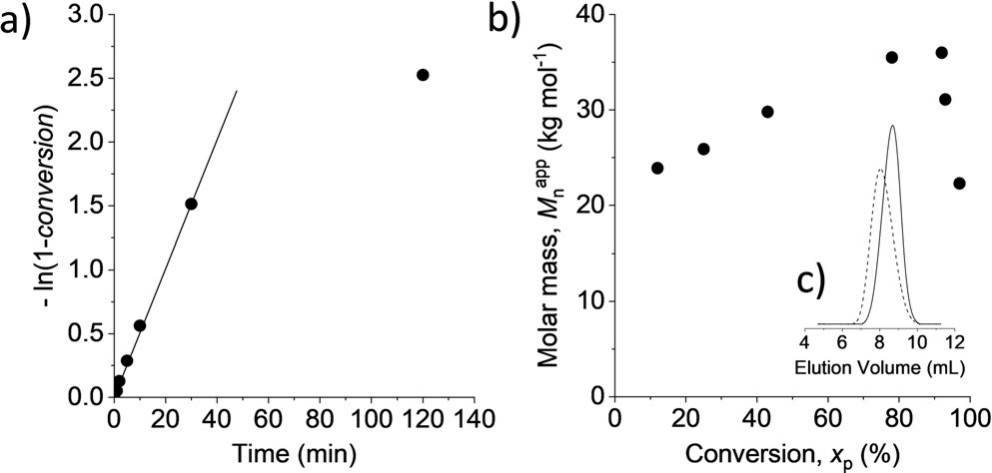
Polymerization of **3** with BF_3_⋅OEt_2_ ([**3**]_o_=4 m, [**3**]_o_/[BF_3_⋅OEt_2_]=10:1) at 0 °C in DCM (entry 12 in Table 2). a) First‐order time‐conversion plot. b) Evolution of number‐average molar mass (M_n_^app^) with monomer conversion (x_p_). c) SEC‐RI trace of the polymer samples (after precipitation into methanol) obtained at 2 h (x_p_=78 %, dashed line) and 24 h (x_p_=97 %, solid line).

As mentioned above, the polymerization did not affect the olefin functionality of the carbohydrate rings. However, we noticed that polymer **4** underwent crosslinking, even when stored at −20 °C, which could be avoided by the exclusion of oxygen or the addition of traces of a radical inhibitor, for example, butylated hydroxytoluene (BHT). It is thought that the allylic ether units in the polymer can form peroxide with atmospheric oxygen,^17^ and this peroxide can potentially act as radical initiator for the olefin crosslinking. Furthermore, the double bonds in **4** are amenable to modification through thiol–ene reactions and hydrogenation, for example (Scheme 3).^18^ The radical additions of methyl 3‐mercaptopropionate using either azobisisobutyronitrile (AIBN) as a radical source at 80 °C or benzophenone/UV light at room temperature were quantitative (polymer **5**), as indicated by the complete disappearance of olefin protons in ^1^H NMR spectra (see the Supporting Information). The hydrogenation of **4** with H_2_/Pd‐C (polymer **6**) was almost quantitative, giving 93 % conversion of double bonds (see the Supporting Information). Spontaneous crosslinking, as earlier observed for **4**, did not happen. The seemingly high reactivity of the 1,2‐disubstituted *cis* olefin towards crosslinking and functionalization makes polymer **4** potentially interesting as a reactive functional material.

**Scheme 3:**
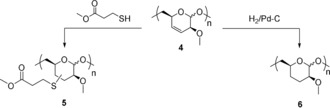
Chemical modification of polymer **4** by radical thiol–ene reaction (left) and by hydrogenation (right).

In summary, levogluconsenyl methyl ether was obtained in two efficient steps from levoglucosenone (derived from cellulose) and successfully polymerized through CROP with either TfOH or BF_3_⋅OEt_2_ to near quantitative conversion. The resulting semicrystalline thermoplastic unsaturated polyacetals exhibited molar masses (*M*_n_^app^) of up to 36 kg mol^−1^ with a dispersity of around 1.4. The polymer readily underwent crosslinking and chemical modification through radical thiol–ene reactions or hydrogenation. This cellulose‐based monomer/polymer system (and derivatives thereof) is potentially interesting to generate a platform of reactive and degradable (co‐)polyacetals or complex macromolecular architectures.^19^ Further studies in this line are in progress, together with optimization of the reaction conditions to achieve a living/controlled (co‐)polymerization, preferably by photochemical processes.

## Conflict of interest

The authors declare no conflict of interest.

## Supporting information

As a service to our authors and readers, this journal provides supporting information supplied by the authors. Such materials are peer reviewed and may be re‐organized for online delivery, but are not copy‐edited or typeset. Technical support issues arising from supporting information (other than missing files) should be addressed to the authors.

Supplementary
