# Supplementary material for: Cellulose‐Derived Functional Polyacetal by Cationic Ring‐Opening Polymerization of Levoglucosenyl Methyl Ether
Source: Angew Chem Int Ed Engl. 2019 Oct 25;58(51):18492–5. doi: 10.1002/anie.201908458 (PMC6916336; doi:10.1002/anie.201908458)
Supplement: Supplementary file 1 — Supplementary [file ANIE-58-18492-s001.pdf]

Angewandte Chemie
Eine Zeitschrift der Gesellschaft Deutscher Chemiker

Supporting Information

Cellulose-Derived Functional Polyacetal by Cationic Ring-Opening Polymerization of Levoglucosenyl Methyl Ether

*Tapas Debsharma, Yusuf Yagci, and Helmut Schlaad**

anie_201908458_sm_miscellaneous_information.pdf

Materials

Levoglucosenone, 99.3% (CAS: 37112-31-5) was purchased from Circa Group Ltd, Australia. Sodium borohydride ($NaBH_4$, 98%), NaH (60% dispersion in oil) and phosphoric acid (H_3PO_4, 98%) were purchased from Acros Chemicals. Benzophenone (99.9%), 10% Pd-C, *sec*-butyl lithium (1.4 M in hexane), *N,N*-dimethylformamide (DMF, 99.99%), CD_2Cl_2 (99.9%), $CDCl_3$ (99.8%), and DMSO-d_6 (99.8%) were purchased from Sigma-Aldrich. Triflic acid (synthesis grade) and $BF_3{\cdot}OEt_2$ were purchased from Merck and Aldrich, respectively. Analytical grade methanol, ethanol, isopropanol, dichloromethane (DCM), tetrahydrofuran (THF), toluene, and acetonitrile were purchased from Fischer Scientific. Azobisisobutyronitrile (AIBN, 98%) was received from Fluka and recrystallized from isopropanol.

Analytical Instrumentation

Nuclear magnetic resonance (NMR) spectra were recorded on Bruker Avance 300 MHz, 500 MHz, or Bruker Avance III 600 MHz spectrometers. The signals were referenced to the solvent peak at δ (^{1}H) 2.50 ppm and (^{13}C) 39.52 ppm for DMSO-d_6, δ (^{1}H) 5.32 ppm and (^{13}C) 53.84 ppm for CD_2Cl_2, and δ (^{1}H) 7.26 ppm and (^{13}C) 77.16 ppm for $CDCl_3$.

Circular dichroism (CD) spectra were recorded on a JASCO J-815 spectrometer at 20 °C, measure range: 290-183 nm, scanning speed: 50 nm min^{-1}, cell length: 1 mm. The sample was dissolved in acetonitrile at a concentration of 0.033 wt%.

Electrospray ionization time-of-flight (ESI-ToF) mass spectrometry was measured in positive ionization mode on a Micromass Q-TOF Micro (Waters Inc.). The sample was dissolved in methanol.

Size exclusion chromatography (SEC) with simultaneous UV and RI (differential refractive index) detection was performed with THF as the eluent (flow rate of 0.5 mL·min^{-1}) at room temperature. The stationary phase was a 300 x 8 mm^2 PSS SDV linear M column (3 µm particle size, molar mass range 10^2-10^6 Da). Solutions containing ~0.15 wt% polymer were filtered through 0.45 µm PTFE filters; the injected volume was 100 µL. Polystyrene standards (PSS, Mainz, Germany) were used for calibration.

Polarized optical microscopy (POM) was performed with an Olympus BX53M polarized optical microscope (crossed polarizers) equipped with a SC50 camera and a Mettler Toledo HS82 hot stage. A polymer film was drop casted on a glass slide and was heated to 120 °C at a heating rate of 20 °C min^{-1}, kept at this temperature for 60 s, and then cooled slowly down to room temperature.

Thermogravimetric analysis (TGA) was measured on a Mettler Toledo TGA/SDTA851 in a temperature range from 25 to 900 °C at a heating rate of 10 °C min^{-1} under continuous nitrogen flow of 20 mL min^{-1}.

Differential scanning calorimetry (DSC) was measured on a Mettler Toledo DSC822e in a temperature range from -50 to 170 °C under continuous nitrogen flow. The heating/cooling rate was 10 or 30 °C min^{-1} (see the temperature profile in Figure S19).

Synthesis of levoglucosenyl methyl ether (3)

NaH dispersed in mineral oil (450 mg considering 60 % NaH) was placed in a dry flask and washed 3 times with dry hexane. Dry THF (30 mL) was added and the dispersion was cooled in an ice bath while constant stirring. To this, a solution of levoglucosenol (**2**)* (10.24 g) in dry THF (30 mL) was slowly added (effervescence was observed). After the effervescence stopped, a solution of methyl iodide (8.8 ml) in dry THF (20 mL) was added and the mixture was allowed to stir at room temperature until thin layer chromatography (TLC) showed complete consumption of the starting material (after about a couple of hours). The crude material was poured into ice-cold water and extracted with DCM several times. The DCM portions were combined and evaporated in a rotary evaporator. The crude compounds were distilled in a Kugelrohr apparatus at 140 °C under reduced pressure (0.1 mbar) to obtain **3** as a colorless oil (overall yield 90%). The levogluconsenyl methyl ether **3** was characterized with the help of ^{1}H, ^{13}C, COSY, and HSQC NMR spectroscopy (Figure S1-S4) and by ESI MS (Figure S5).

* Synthesis of levoglucosenol (**2**) by reduction of levoglucosenone (**1**), see *Angew. Chem. Int Ed.* **2019**, *58 (20)*, 6718-6721, SI.

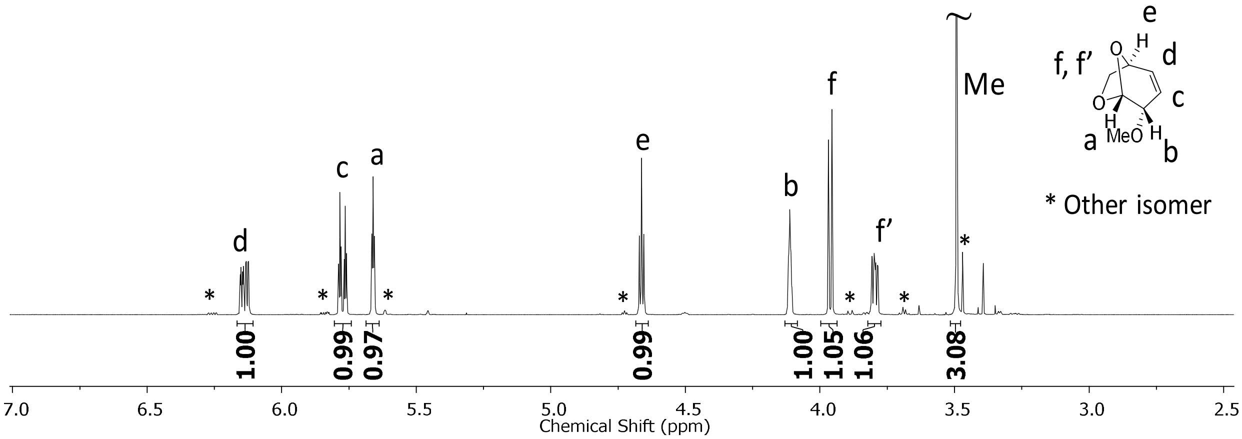

Figure S1. ^{1}H NMR (500 MHz, $CDCl_3$) spectrum of **3**; δ 6.14 (ddd, *J* = 9.9, 4.2, 1.2 Hz, 1H), 5.77 (dt, *J* = 10.0, 2.2 Hz, 1H), 5.66 (t, *J* = 2.3 Hz, 1H), 4.66 (t, *J* = 4.1 Hz, 1H), 4.12-4.10 (m, 1H), 3.96 (d, *J* = 6.6 Hz, 1H), 3.80 (ddd, *J* = 6.5, 4.1, 1.1 Hz, 1H), 3.49 (s, 3H).

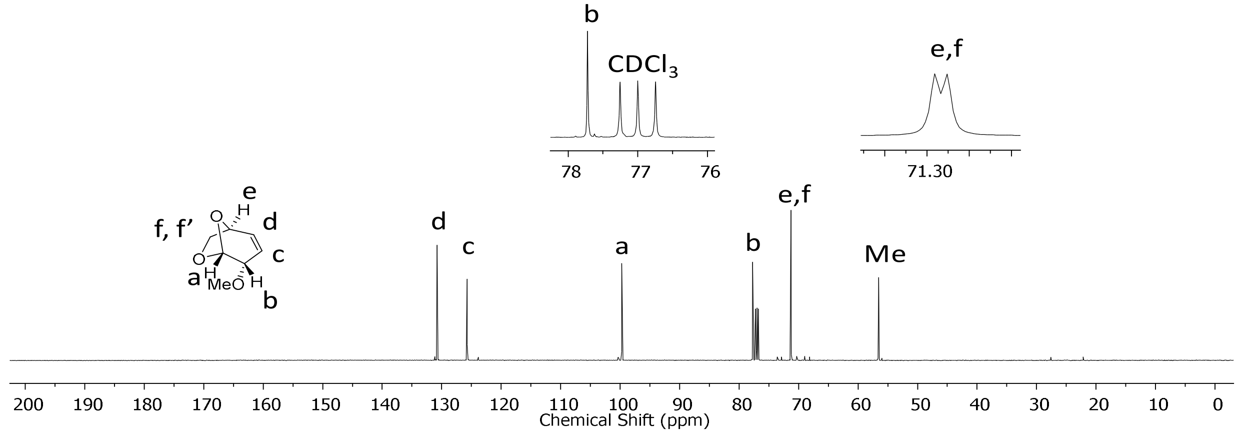

Figure S2: ^{13}C NMR (125 MHz, $CDCl_3$) spectrum of **3**; δ 130.8, 125.7, 99.7, 77.7, 71.3, 71.3, 56.6.

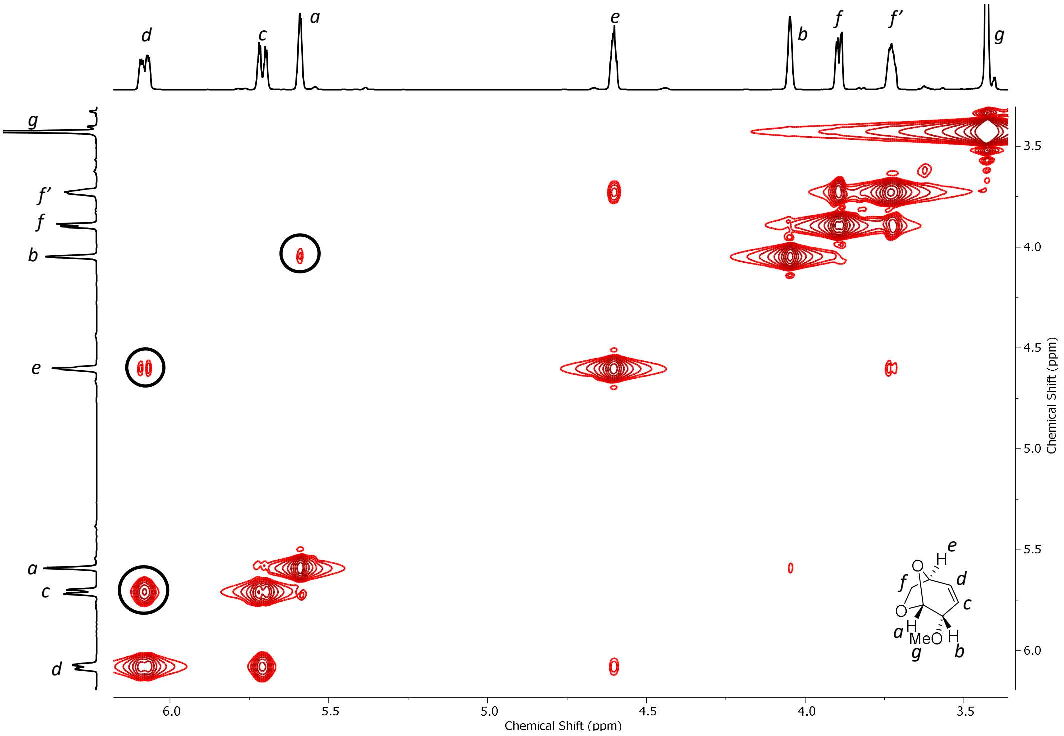

Figure S3: $^{1}H,^{1}H$-COSY NMR (500 MHz, $CDCl_3$) spectrum of **3**.

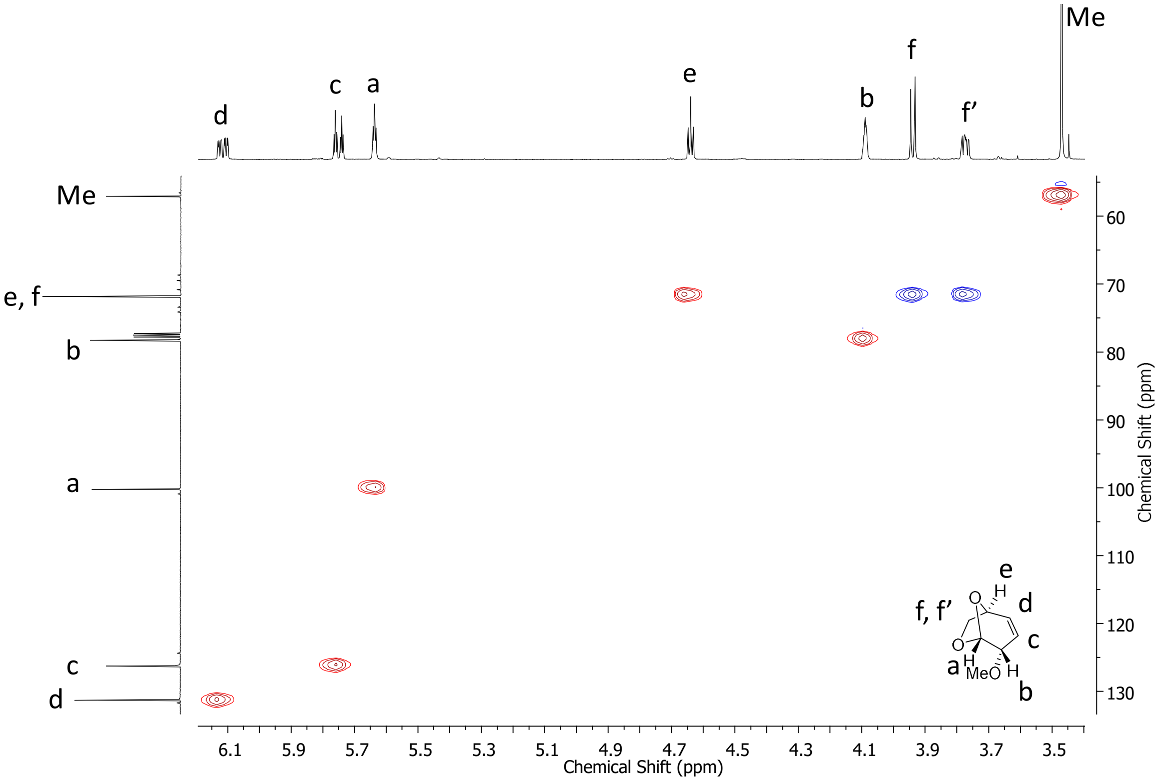

Figure S4: HSQC NMR (125 MHz, 500 MHz, $CDCl_3$) spectrum of **3** (blue: negative phase, CH_2; red: positive phase, CH and CH_3).

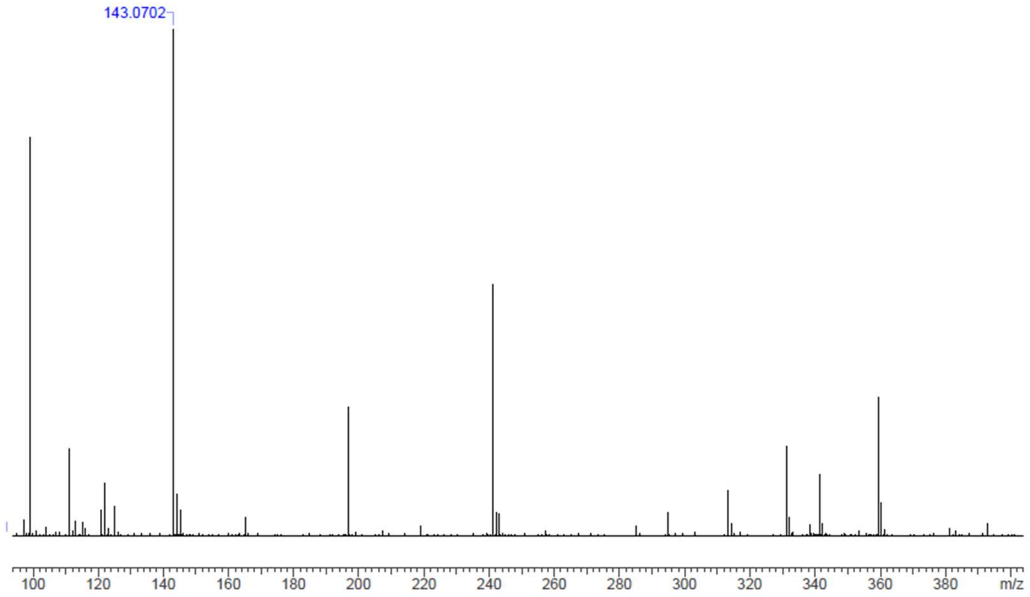

Figure S5: ESI MS spectrum of **3**; m/z 143.0702 (calculated: 143.070).

Polymerization - general procedure

In a typical experiment, 2 mmol of **3** were dissolved in a certain amount of dry DCM (depending on targeted concentration) and kept in a temperature bath (depending on desired temperature). A required amount of stock solution of the acid initiator/catalyst (TfOH or $BF_3{\cdot}OEt_2$) in dry DCM was added and the mixture was stirred for a pre-determined time. The reaction was quenched with an excess of triethylamine followed by precipitation of the polymer (**4**) into methanol. The polymer was centrifuged and dried in a vacuum oven at 40 °C.

Table S1. Polymerization of **3** with TfOH in dry DCM solution under various reaction conditions.

Run	[**3**]$_o$/[TfOH]	[**3**]$_o$ (M)	T (°C)	*Time* (h)	x_p [a] (%)	M_n^{app} [b] (kg/mol)	$Đ$ [b]
1	200:1	4	25	24	92	15.1	1.4
2	200:1	4	0	24	92	18.6	1.4

[a] Monomer conversion by ^{1}H NMR spectroscopy. [b] Number-average molar mass (M_n^{app}) and dispersity ($Đ$) by SEC with polystyrene calibration.

Table S2. Polymerization of **3** with $BF_3{\cdot}OEt_2$ in dry DCM solution under various reaction conditions.

Run	[**3**]$_o$/ [$BF_3{\cdot}OEt_2$]	[**3**]$_o$ (M)	T (°C)	Time (h)	Appearance	x_p [a] (%)	M_n^{app} [b] (kg/mol)	$Đ$ [b]
1	100:10	1	-50	48	Liquid	n.d.	n.d.	n.d.
2	100:10	2	-50	48	Liquid	n.d.	n.d.	n.d.
3	100:10	3	-50	48	Viscous Liquid	3	n.d.	n.d.
4	100:10	4	-50	48	Liquid → Solid[c]	9	18.6	1.33
5	100:10	1	-20	48	Liquid	19	n.d.	n.d.
6	100:10	2	-20	48	Liquid	57	23.2	1.25
7	100:10	3	-20	48	Viscous Liquid	70	23.9	1.29
9	100:10	4	-20	24	Liquid → Solid[c]	74	25.3	1.31
10	100:10	2	-10	24	Liquid	68	18.6	1.27
11	100:10	3	-10	24	Viscous Liquid	77	19.3	1.33
13	100:10	4	-10	24	Viscous Liquid	87	21.2	1.43
14	100:20	1	-10	24	Liquid	61	13.0	1.47
15	100:20	2	-10	24	Liquid	85	17.6	1.44
16	100:20	3	-10	24	Viscous Liquid	92	20.3	1.44
17	100:20	4	-10	24	Viscous Liquid	94	21.7	1.52
18	100:30	4	-10	24	Viscous Liquid	97	28.8	1.43
19	100:10	2	0	24	Liquid	85	14.6	1.31
20	100:10	3	0	24	Viscous Liquid	90	17.2	1.36
21	100:10	4	0	24	Viscous Liquid	93	19.8	1.39
22	200:01	2	24	2	Liquid	2	n.d.	n.d.
23	100:20	2	0	2	Liquid	84	11.8	1.46
24	100:20	2	0	24	Liquid	85	14.7	1.28

[a] Monomer conversion by ^{1}H NMR spectroscopy. [b] Number-average molar mass (M_n^{app}) and dispersity ($Đ$) by SEC with polystyrene calibration. [c] Solid dissolved upon dilution.

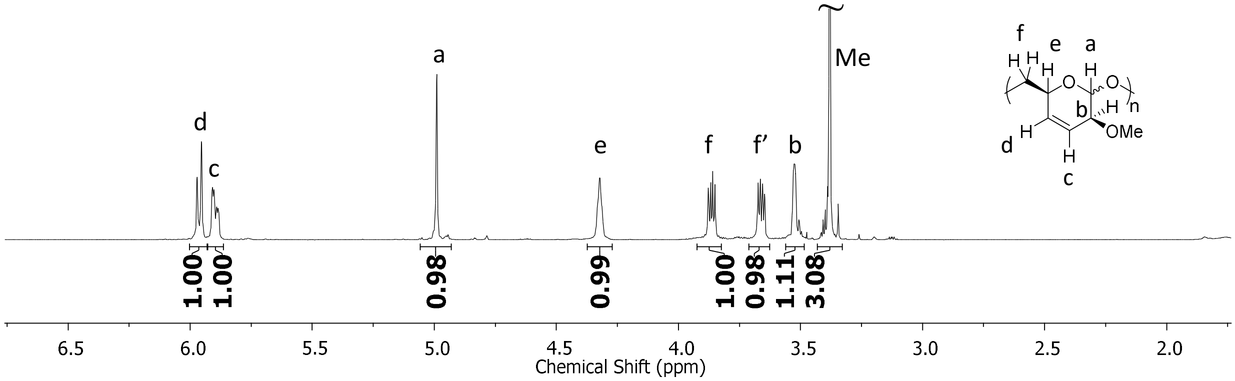

Figure S6. ^{1}H NMR (600 MHz, $CDCl_3$) spectrum of polymer **4** (entry 11 in Table S2); 6.11 – 5.92 (m, 1H), 5.93 – 5.83 (m, 1H), 4.99 (s, 1H), 4.36 – 4.27 (m, 1H), 3.92 – 3.82 (s, 1H), 3.67 (dd, J = 10.7, 5.0 Hz, 1H), 3.61 – 3.50 (m, 1H), 3.38 (s, 3H).

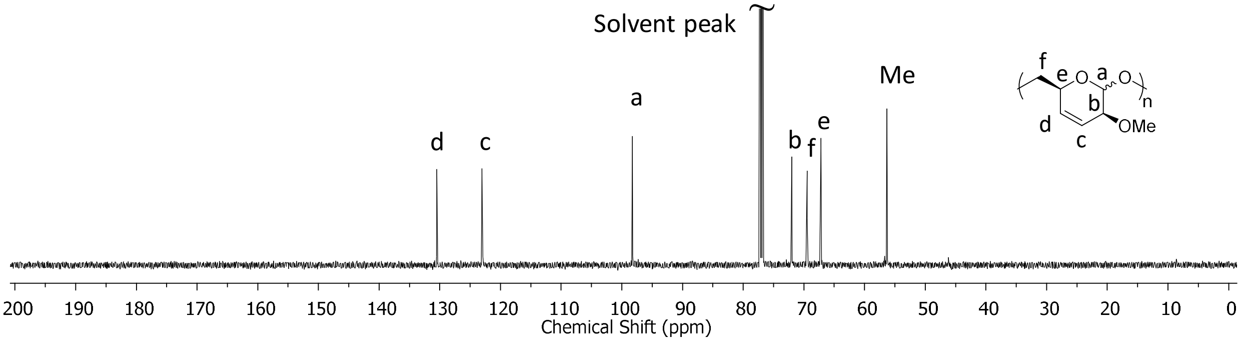

Figure S7. ^{13}C NMR (150 MHz, $CDCl_3$) spectrum of **4** (entry 11 in Table S2); δ 130.5, 123.0, 98.3, 72.0, 69.5, 67.2, 56.3

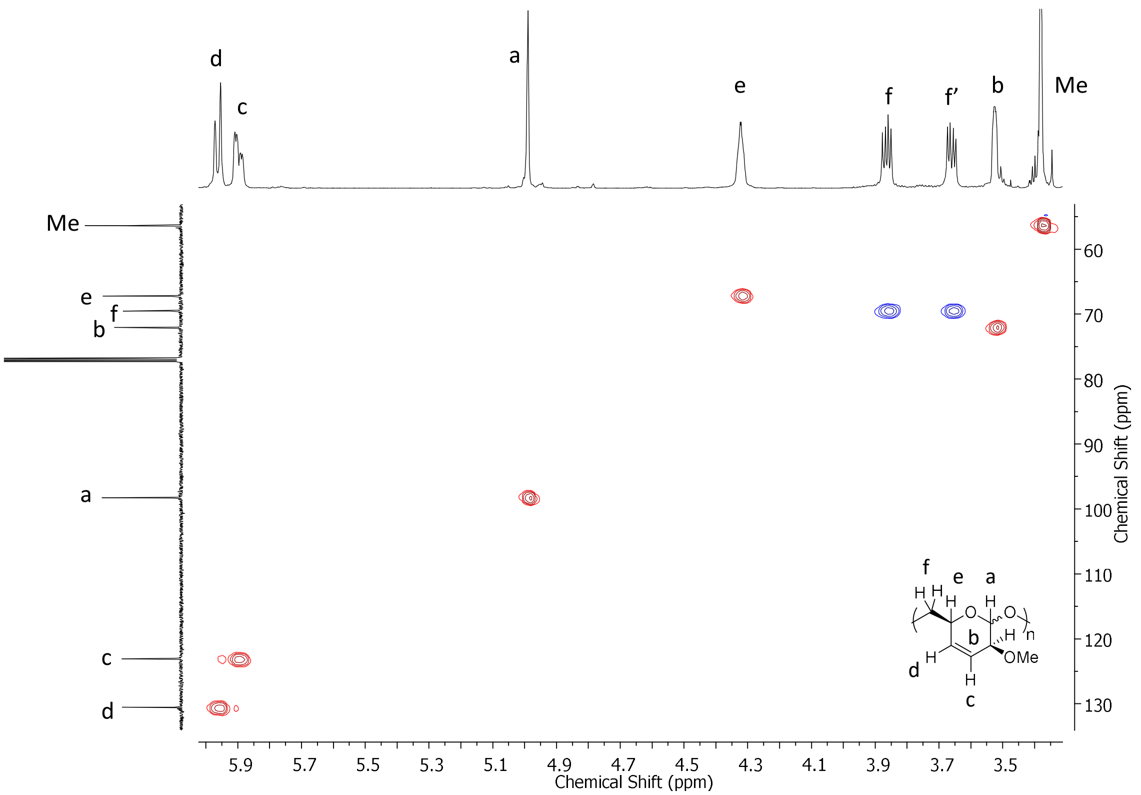

Figure S8. HSQC NMR (150 MHz, 600 MHz, $CDCl_3$) spectrum of polymer **4** (entry 11 in Table S2).

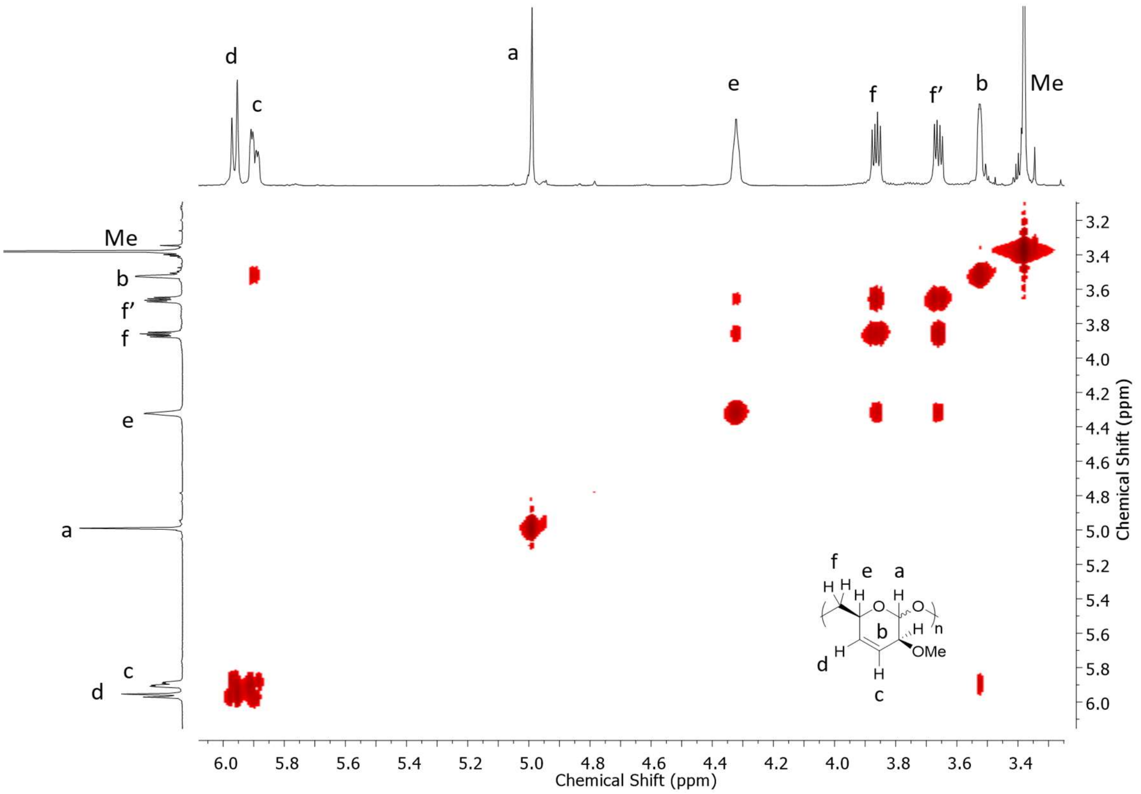

Figure S9. COSY NMR (600 MHz, 600 MHz, $CDCl_3$) spectrum of polymer **4** (entry 11 in Table S2).

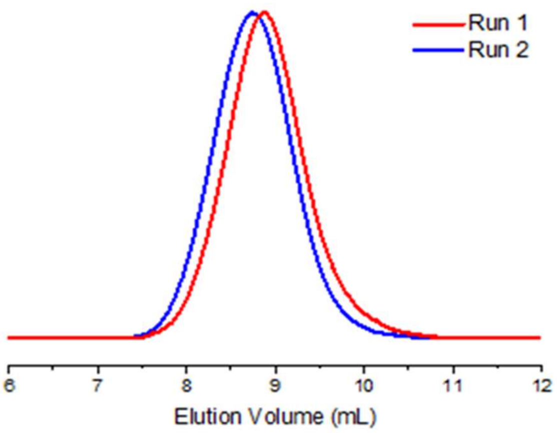

Figure S10a. SEC-RI traces of polymer **4**, entries 1-2 in Table S1 (same in Table 1)**.**

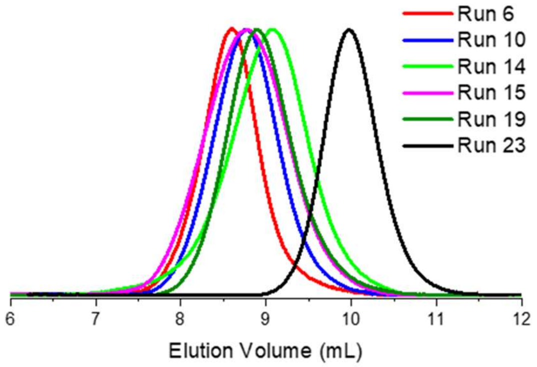

Figure S10b. SEC-RI traces of polymer **4**, entries 6, 10, 14, 15, 19, and 23 in Table S2.

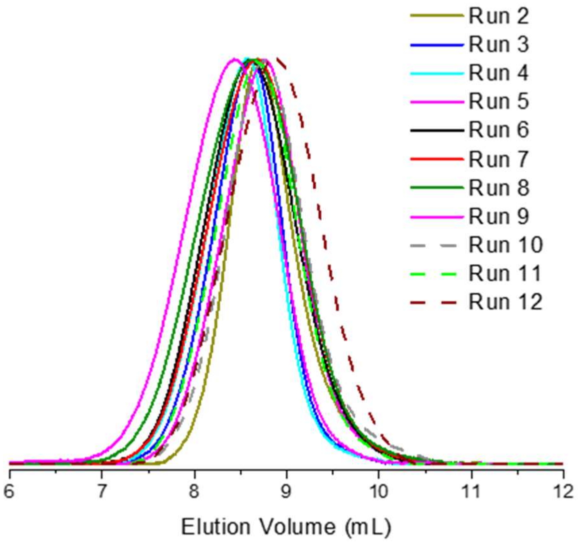

Figure S10c. SEC-RI traces of polymer **4**, entries 2-12 in Table 2.

Kinetic investigation

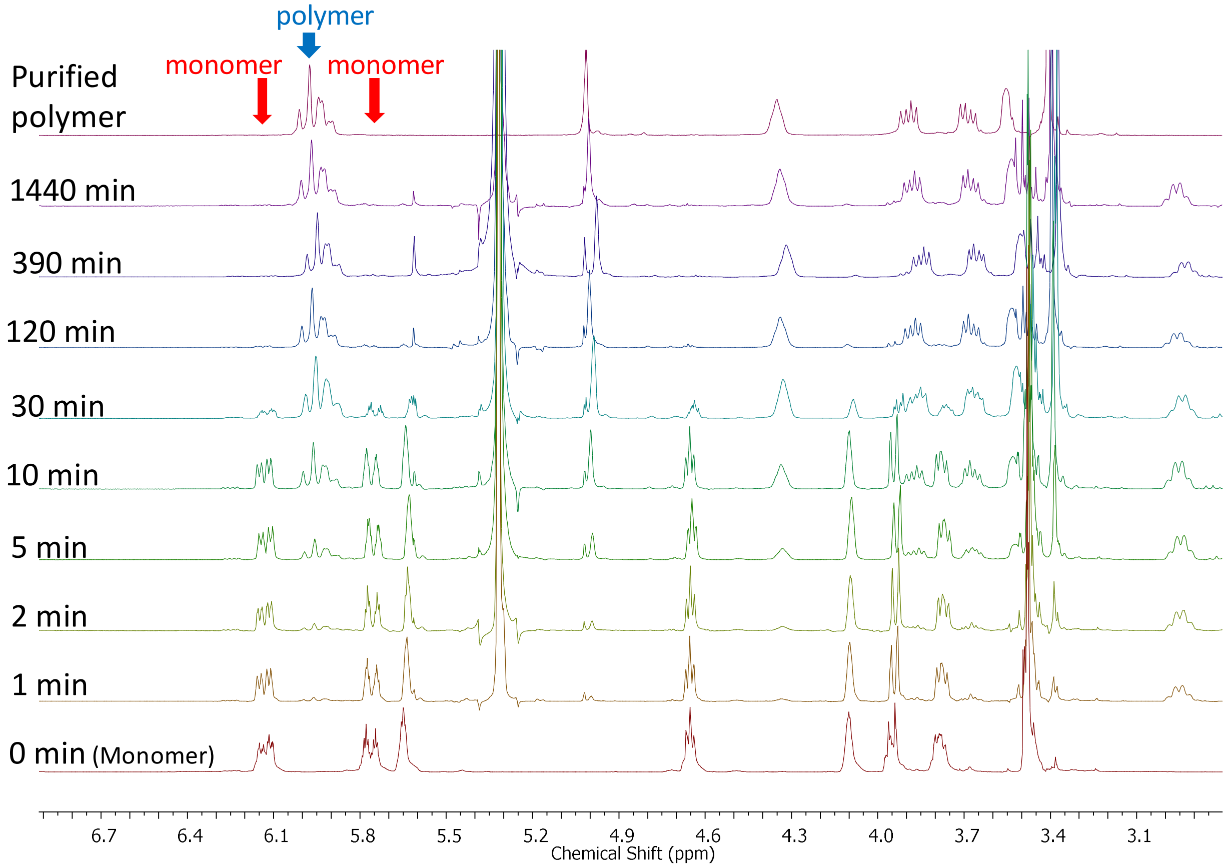

Figure S11. Kinetic study of the polymerization of **3** with $BF_3{\cdot}OEt_2$ at 0 °C by ^1H NMR (300 MHz, $CDCl_3$). The olefinic protons at δ 5.7-6.2 were used to determine the monomer conversion.

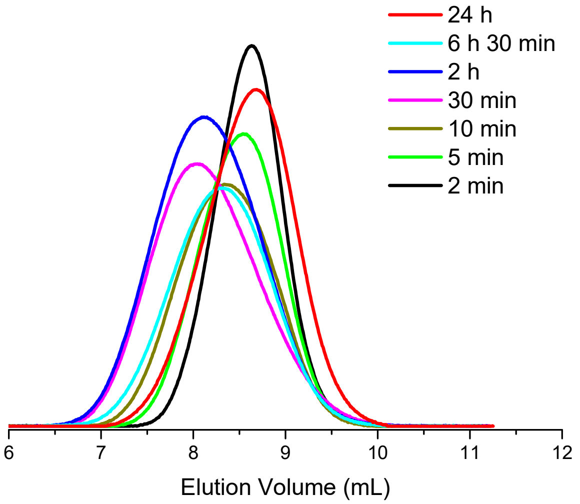

Figure S12. SEC-RI traces of the polymers **4** obtained at different time intervals during the polymerization of **3** with $BF_3{\cdot}OEt_2$ at 0 °C (the sample obtained after 1 min did not precipitate).

Thiol-ene addition

(1) Initiation by UV: 50 mg of the polymer **4** and 64 mg of benzophenone were placed in a 9 ml septum sealed transparent vial. Then, 420 mg of methyl 3-mercaptopropionate and 1 mL of dry THF were added. After three freeze-pump-thaw cycles, the mixture was irradiated with a 150 W Hg-medium pressure UV lamp for 18 h. The polymer (**5**) was then precipitated into methanol, centrifuged, and dried in a vacuum oven at 40 °C overnight.

(2) Initiation by AIBN: 50 mg of the polymer **4** and 164 mg of AIBN were placed in a 9 ml septum sealed transparent vial. Then, 420 mg of methyl 3-mercaptopropionate. After three freeze-pump-thaw cycles, the mixture was heated at 80 °C for 18 h. The polymer (**5**) was then precipitated into methanol, centrifuged, and dried in a vacuum oven at 40 °C overnight.

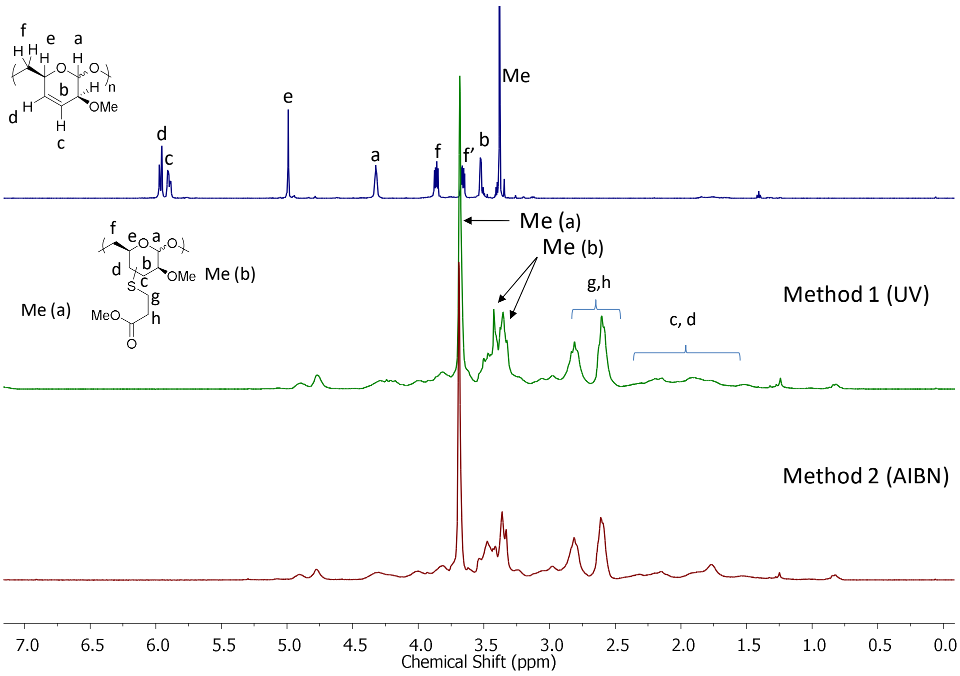

Figure S13. ^{1}H NMR spectra of polymer **5** (300 MHz, $CDCl_3$) obtained by thiol-ene methods (1) (UV) and (2) (AIBN) and of the precursor polymer **4** (600 MHz, $CDCl_3$)

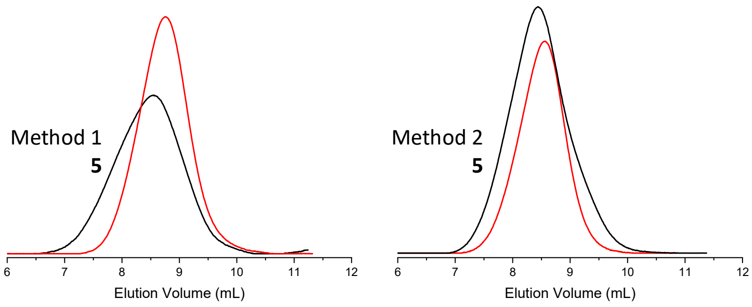

Figure S14. SEC-RI traces of polymer **4** (red) and polymer **5** (black) obtained by thiol-ene method (1, UV) (left) and method (2, AIBN) (right).

Hydrogenation

50 mg of polymer **4** was dissolved in 5 mL of THF and 1 mL of hexane, and 5 mg of 10% Pd-C was added. The mixture was subjected to H_2 gas at atmospheric pressure for 7 days. The polymer **6** was precipitated into methanol, centrifuged, and dried in a vacuum oven at 40 °C.

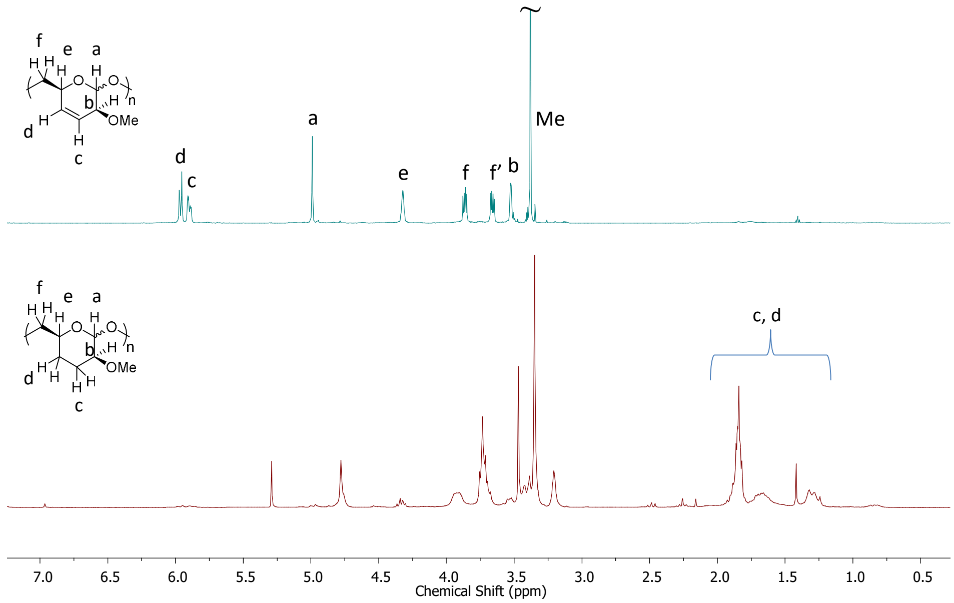

Figure S15. ^{1}H NMR spectra of the precursor polymer **4** (600 MHz, $CDCl_3$) (top) and the hydrogenated polymer **6** (300 MHz, $CDCl_3$) (bottom).

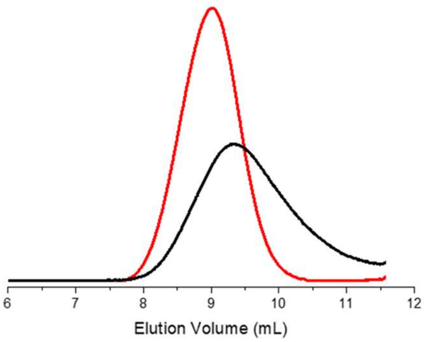

Figure S16. SEC-RI traces of polymer **4** (red) and hydrogenated polymer **6** (black).

Degradation

To a solution of polymer 4 (40 mg, 0.29 mmol) in 1 mL of DCM were added two drops of methanol and 0.23 mL (1.82 mmol) of $BF_3{\cdot}OEt_2$. The mixture was stirred for 10 h at room temperature, quenched with triethylamine, evaporated to dryness, and analyzed by SEC.

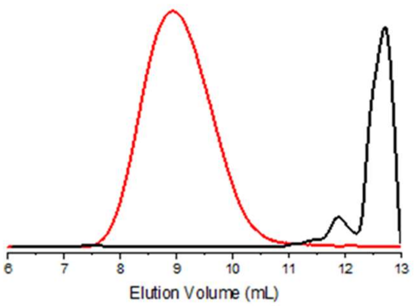

Figure S17. SEC-RI traces of polymer **4** (red) and the sample obtained after 10 h treatment with methanol/$BF_3{\cdot}OEt_2$ (black).

Thermal analysis of polymer 4

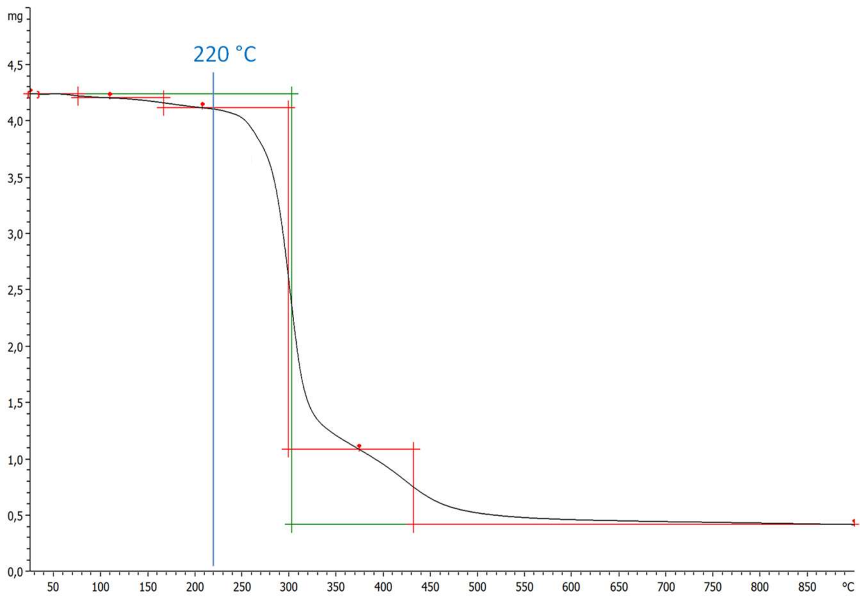

Figure S18. Exemplary TGA curve of polymer **4** (entry 12 in Table 2).

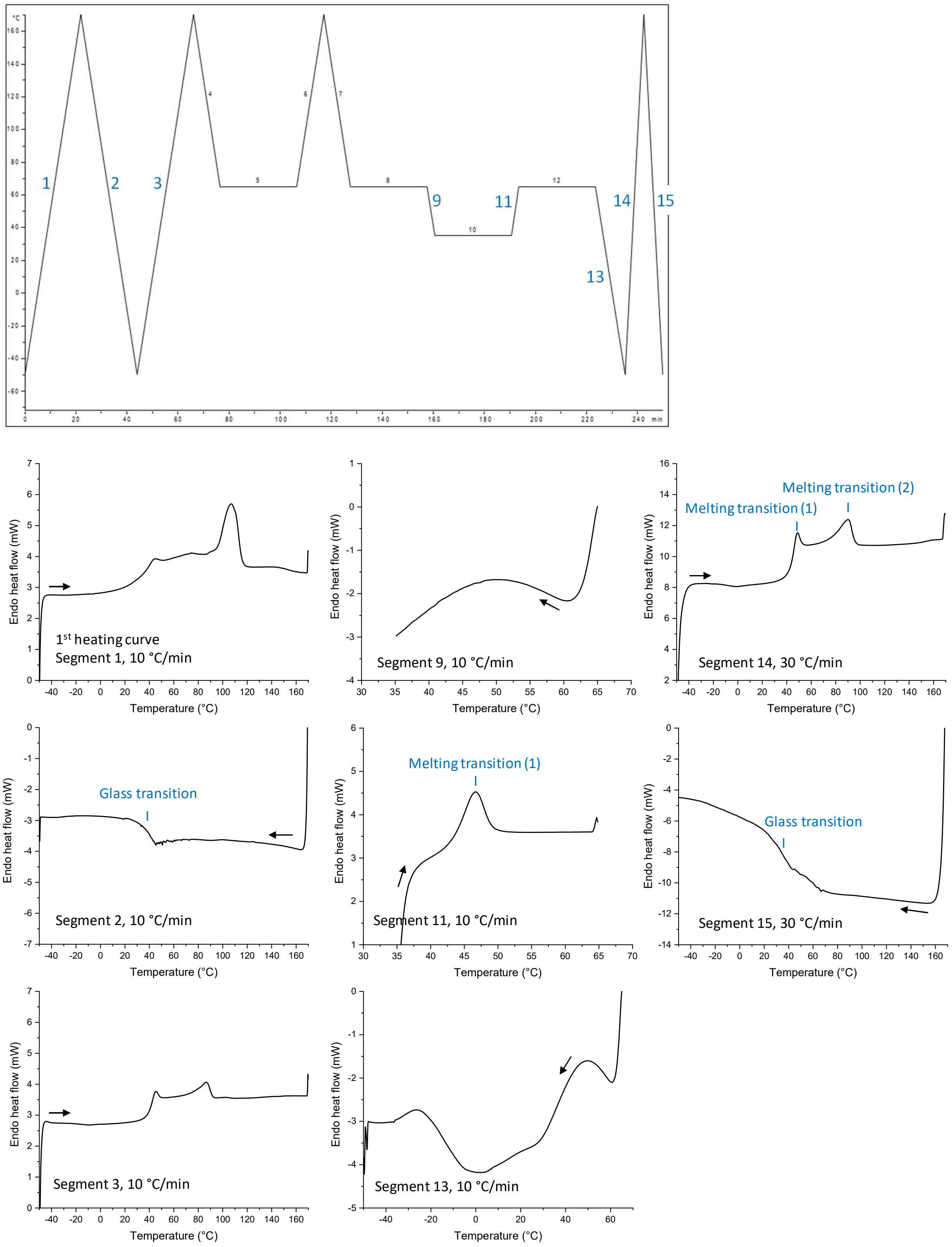

Figure S19. Selected DSC curves (bottom) obtained for polymer **4** (entry 12 in Table 2) following the above shown temperature profile (top).
